# Blood-based extracellular matrix biomarkers as predictors of survival in patients with metastatic pancreatic ductal adenocarcinoma receiving pegvorhyaluronidase alfa

**DOI:** 10.1186/s12967-021-02701-z

**Published:** 2021-01-21

**Authors:** Song Wang, Cecilie L. Bager, Morten A. Karsdal, Dimitrios Chondros, Darin Taverna, Nicholas Willumsen

**Affiliations:** 1 Halozyme Therapeutics, Inc., San Diego, CA USA; 2 Nordic Bioscience A/S, Herlev Hovedgade 207, 2730 Herlev, Denmark

**Keywords:** Biomarkers, Plasma, Collagen, Pegvorhyaluronidase alfa, PEGPH20, Pancreatic ductal adenocarcinoma, Extracellular matrix, Hyaluronan, Stroma modifier

## Abstract

**Background:**

Extensive extracellular matrix (ECM) remodeling is a hallmark of metastatic pancreatic ductal adenocarcinoma (mPDA). We investigated fragments of collagen types III (C3M, PRO-C3), VI (PRO-C6), and VIII (C8-C), and versican (VCANM) in plasma as biomarkers for predicting progression-free survival (PFS) and overall survival (OS) in patients with mPDA treated with pegvorhyaluronidase alfa, a biologic that degrades the ECM component hyaluronan (HA), in a randomized phase 2 study (HALO109-202).

**Methods:**

HALO109-202 comprised a discovery cohort (Stage 1, n = 94) and a validation cohort (Stage 2, n = 95). Plasma ECM biomarkers were analyzed by ELISAs. Univariate Cox regression analysis and Kaplan–Meier plots evaluated predictive associations between biomarkers, PFS and OS in patients treated with pegvorhyaluronidase alfa plus nab-paclitaxel/gemcitabine (PAG) versus nab-paclitaxel/gemcitabine (AG) alone.

**Results:**

PFS was improved with PAG vs. AG in Stage 1 patients with high C3M/PRO-C3 ratio (median cut-off): median PFS (mPFS) 8.0 vs. 5.3 months, *P *= 0.031; HR = 0.40; 95% CI 0.17–0.92). High C3M/PRO-C3 ratio was validated in Stage 2 patients by predicting a PFS benefit of PAG vs. AG (mPFS: 8.8 vs. 3.4 months, *P *= 0.046; HR = 0.46; 95% CI 0.21–0.98). OS was also improved in patients with high C3M/PRO-C3 ratio treated with PAG vs. AG (mOS 13.8 vs 8.5 months, *P *= 0.009; HR = 0.35; 95% CI 0.16–0.77). Interestingly, high C3M/PRO-C3 ratio predicted for a PFS benefit to PAG vs. AG both in patients with HA-low tumors (HR = 0.36; 95% CI 0.17–0.79) and HA-high tumors (HR = 0.20; 95% CI 0.06–0.69).

**Conclusions:**

The C3M/PRO-C3 ratio measuring type III collagen turnover in plasma has potential as a blood-based predictive biomarker in patients with mPDA and provides additional value to a HA biopsy when applied for patient selection.

*Trial registration:* NCT01839487. Registered 25 April 2016

## Background

Pancreatic cancer has one of the worst prognoses of all major cancers, with 5-year survival rates as low as 8% overall and 3% for Stage 4 disease [1]. Low survival rates reflect few early detection tools and limited efficacy of available therapies [2]. Pancreatic ductal adenocarcinoma (PDA), the most common type of pancreatic cancer, is largely resistant to systemic therapies, partly due to excessive accumulation of collagen, versican (VCANM), hyaluronan (HA), which form a dense desmoplasia composed also of extra-cellular matrix (ECM) proteins, myofibroblastic-like pancreatic stellate cells and immune cells [3, 4]. This unique tumor microenvironment (TME), characterized by growth of dense, collagen-rich ECM and stroma around cancer cells (the desmoplastic reaction), promotes tumor growth and metastasis, forming a physical barrier to systemic therapies.

Accumulated HA molecules in the TME in PDA are able to complex large amounts of water to create a sizable, less mobile gel-fluid phase, increasing tumor interstitial pressure, leading to compressed tumor vasculature, hypoxia, and reduced access and activity of chemo- and immunotherapies, and infiltration of immune cells [5–11]. The HA-rich desmoplastic TME promotes tumor progression by enhancing host–tumor interactions, such as tumor cell proliferation and angiogenesis [12]. HA binds and interacts with VCANM, and HA overproduction in presence of VCANM can accelerate angiogenesis [13]. Accumulation of HA in tumors also affects ECM protein turnover [14]. This increased HA content has been associated with elevated collagen and alpha-smooth muscle actin (αSMA) levels in tumors [15]. Indeed, HA and the major collagen types (I, III, and IV) accumulate in the stroma of primary and metastatic pancreatic tumors compared with adjacent normal tissue [9]. Several studies link HA with collagen synthesis in wound healing, where exogenous HA leads to significantly increased expression of type III collagen, high molecular weight HA leads to increased collagen type III and HA fragments increase collagen type I deposition [16, 17]. Furthermore, HA is involved in type VI collagen synthesis and structural integrity, it induces type VIII collagen production, which is involved in angiogenesis and artery remodeling, and type I collagen in vitro [18–21].

During cancer progression, ECM undergoes excessive protein turnover, including collagen formation and remodeling/degradation by matrix metalloproteinases (MMP) [22]. Consequently, increased levels of tissue and cancer-specific ECM turnover products (ECM neo-peptides) are released into the circulation, including specific fragments of MMP-mediated degradation of type I, III and IV collagen (C1M, C3M, C4M), and type III and VI collagen formation (pro-peptides PRO-C3 and PRO-C6) [23–27]. MMP-generated fragments are indicative of ECM degradation, while specific collagen pro-peptides are suggestive of collagen formation, providing surrogate measures of tissue turnover/disease activity [22]. ECM neo–peptides are increasingly investigated as potential predictive and prognostic biomarkers. For example, C3M and PRO-C3 have shown prognostic and predictive value in metastatic breast cancer and pancreas cancer [28–30]. Furthermore, a high ratio of type III collagen degradation to formation (C3M/PRO-C3) at baseline predicts overall survival (OS) in patients with metastatic melanoma and advanced pancreas cancer [23, 30]. Direct measurements of collagen type VIII using an antibody raised to the C-terminal (C8-C), or analysis of expression levels of versican/VCANM have also been used as biomarkers in several cancers [20, 31–34].

Pegvorhyaluronidase alfa (PEGPH20; PVHA) is first-in-class biologic that enzymatically degrades tumor HA, reducing tumor interstitial pressure and improving vascular perfusion, thereby decreasing hypoxia, and increasing the access and efficacy of anti-cancer therapies as well as infiltration of immune cells [6–8, 11, 15]. Results of a randomized phase 2 study (HALO 109-202; NCT01839487), demonstrated promising activity of pegvorhyaluronidase alfa in combination with nab–paclitaxel and gemcitabine (PAG) in patients with metastatic PDA (mPDA) [35]. Median progression–free survival (PFS) improved significantly with PAG versus nab-paclitaxel plus gemcitabine (AG; 6.0 months versus 5.3 months; hazard ratio [HR] 0.73; 95% confidence interval [CI] 0.53–1.00; *P* = .049). The improvement in median PFS was more pronounced in patients with HA-high tumors, identified by an affinity histochemistry assay (HA diagnostic RxDx; Ventana Medical Systems, Inc., Tucson, AZ, USA and Halozyme Therapeutics, Inc., San Diego, CA, USA); 9.2 months with PAG versus 5.2 months with AG; HR 0.51; 95% CI 0.26–1.00; *P* = .048), suggesting that HA staining in tumor biopsies was a potential biomarker for selecting patients most likely benefiting from this treatment. Although the phase 2 study showed promising results in patients with HA-high tumors, the phase 3 study HALO109-301 study failed to meet its primary endpoint by including only patients with HA-high tumors and further development of pegvorhyaluronidase alfa was halted [36, 37]. This warrants further investigations into the molecular biology and validation of novel biomarkers to better characterize the complexity of the TME for predicting response to stromal modifiers in the mPDA setting.

Liquid biomarkers that predict response to treatment could spare patients from unnecessary invasive procedures and may provide benefit when tissue biopsies are difficult to obtain [38]. Using data from the HALO109-202 study, we investigated whether plasma circulating biomarkers of ECM remodeling, i.e., PRO-C3, PRO-C6, C3M, C8-C, and VCANM correlated with survival outcomes in patients with mPDA following pegvorhyaluronidase alfa treatment, and could be used as biomarkers of response.

## Methods

### Study design

HALO 109-202 was a phase 2, randomized, multicenter study (NCT01839487) comparing efficacy and safety of PAG versus AG in patients with Stage 4 mPDA who had no prior treatment for metastatic disease, and a life expectancy of ≥ 3 months.

The study has been described in detail elsewhere, Hingorani et al. [35]. In the present study, only patients with sufficient plasma samples for biomarker analysis and efficacy data were included which differs from the inclusion criteria described by Hingorani et al., where a plasma sample were not needed. Briefly, HALO 109-202 was initiated in March 2013 and was placed on brief clinical hold in April 2014 due to an imbalance in thromboembolic (TE) events observed between treatment arms. The study resumed in August 2014, after the protocol was amended to include daily thromboprophylaxis with enoxaparin in both treatment arms and exclude patients with prior TE events. The study comprised two stages: in Stage 1 (prior to clinical hold), patients were randomized 1:1 to receive PAG or AG; in Stage 2 (after clinical hold was lifted), patients were randomized 2:1 to receive PAG or AG. Here, we report additional results from the December 2016 data cut-off point.

### Assessment of biomarker concentrations

An exploratory objective of the HALO 109-202 study was to identify potential biomarkers to predict outcomes of pegvorhyaluronidase alfa treatment. Stage 1 was used as a discovery cohort for biomarkers, and Stage 2 as a validation cohort.

Plasma samples were collected from patients with available baseline assessments (defined as any plasma sample collected before pegvorhyaluronidase alfa administration on Cycle 1, day 1) during Stage 1 and Stage 2. Samples were preserved in K3-ethylenediaminetetraacetic acid (EDTA) and stored at − 70 °C under continuous temperature monitoring. Concentrations of neo-peptides of key ECM proteins, including collagen types III (C3M (cat. no. 1200-02), PRO-C3 (cat. no. 1700-05), VI (PRO-C6 (cat.no. 4000)), and VIII (C8-C (cat. no. 8000)), and VCANM (cat. no. 6000) (Additional file 1: Table S1 and Fig. S1), were measured by a technician in a blinded manner using highly specific competitive enzyme-linked immunosorbent assays, per manufacturer’s instructions (Nordic Bioscience A/S, Herlev, Denmark). The original technical evaluation of each assay is described elsewhere [20, 39–42]. The optical density was measured at 450 nm with 650 nm as reference. A four-parametric fitted model was used to generate a standard curve. Data were analyzed using SoftMax Pro v.6.3 (Molecular Devices, LLC, San Jose, CA, USA).

### Outcomes

Association between baseline concentrations of circulating ECM neo-peptides and PFS, OS, and objective response rate (ORR), defined by the Response Evaluation Criteria in Solid Tumors v1.1, was assessed.

### Statistical analysis

Sample size was determined in a retrospective manner and was not designed to detect a specified effect size for biomarkers. Univariate Cox proportional-hazards regression models were used to evaluate the predictive value of biomarkers for PFS and OS in response to treatment (PAG versus AG). Median and receiver-operating characteristic (ROC) cut-off points (described below) were used to define biomarker-high and biomarker-low patient groups. Survival outcomes were reported as forest plots and Kaplan–Meier survival plots compared by log-rank test. Data for the ORR endpoint were analyzed descriptively, as the number of responders was too small for statistical analyses.

The two cut-off points used to demonstrate the predictive value of biomarkers were informed by two different approaches. The median cut-off was determined from the biomarker values alone as the median value measured for each biomarker in patients from Stage 1 of the study. The ROC cut-off was derived from the best overall response (BOR) data being combined with the biomarker data by ROC curve analysis of patients receiving PAG in Stage 1, with the classification variables: progressive disease compared with complete response + partial response + stable disease. A ROC curve of sensitivity plotted against (1 − specificity) was constructed using the various biomarker values. Youden’s index (J = sensitivity + specificity − 1) was calculated for all points on the ROC curve. The maximum value of Youden’s index was selected as the optimal ROC cut-off, which optimizes a biomarker’s differentiating ability when equal weight is given to sensitivity and specificity [43]. A significance level of *P* < .05 was used. Statistical analysis was performed using MedCalc version 16.8.4 (MedCalc Software bvba, RRID:SCR_015044, Ostend, Belgium).

## Results

### Predictive value of ECM biomarkers for pegvorhyaluronidase alfa treatment

**Fig. 1:**
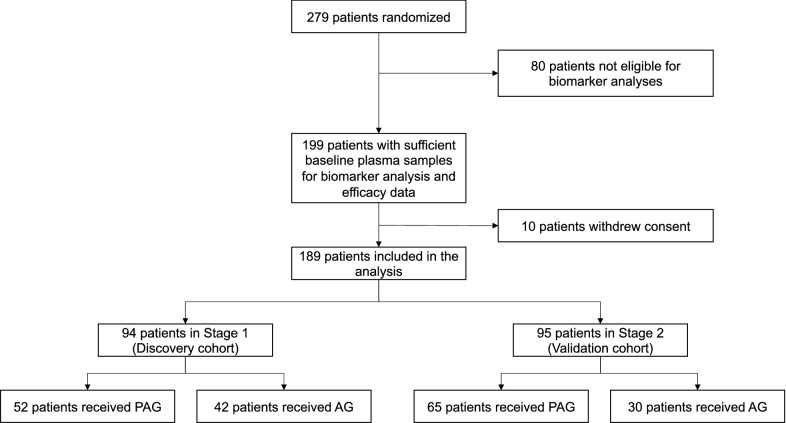
Flow of patients in the HALO 109-202 plasma biomarker analysis

Among 279 patients randomized into the HALO 109-202 study, 199 had both sufficient plasma samples for biomarker analysis and efficacy data; of these, 10 withdrew consent. Baseline plasma samples were therefore available for analysis from 189 patients (Fig. 1). Baseline demographics for these patients are provided in Table 1.

**Table 1:** Baseline Characteristics by Stage (Discovery/Validation Cohort)

Characteristics	Stage 1 (discovery cohort)			Stage 2 (validation cohort)		
	PAG (N = 52)	AG (N = 42)	Total (N = 94)	PAG (N = 65)	AG (N = 30)	Total (N = 95)
Mean age (min–max), years	62.5 (28–77)	66.8 (54–76)	64.4 (28–77)	64.7 (40–83)	63.3 (44–80)	64.3 (40–83)
Sex, n (%)						
Female	19 (36.5)	23 (54.8)	42 (44.7)	26 (40.0)	16 (53.3)	42 (44.2)
Male	33 (63.5)	19 (45.2)	52 (55.3)	39 (60.0)	14 (46.7)	53 (55.8)
Race, n (%)						
White	47 (90.4)	33 (78.6)	80 (85.1)	58 (89.2)	24 (80.0)	82 (86.3)
Black or African American	3 (5.8)	7 (16.7)	10 (10.6)	4 (6.2)	1 (3.3)	5 (5.3)
Asian	0 (0.0)	1 (2.4)	1 (1.1)	2 (3.1)	3 (10.0)	5 (5.3)
Multiple	0 (0.0)	1 (2.4)	1 (1.1)	0 (0.0)	0 (0.0)	0 (0.0)
American Indian or Alaska Native	1 (1.9)	0 (0.0)	1 (1.1)	0 (0.0)	0 (0.0)	0 (0,0)
Native Hawaiian or Other Pacific Islander	1 (1.9)	0 (0.0)	1 (1.1)	0 (0.0)	0 (0.0)	0 (0.0)
Other	0 (0.0)	0 (0.0)	0 (0.0)	0 (0.0)	2 (6.7)	2 (2.1)
Primary tumor location (pancreas), n (%)						
Body	26 (50.0)	16 (38.1)	42 (44.7)	26 (40.0)	12 (40.0)	38 (40.0)
Head	16 (30.8)	17 (40.5)	33 (35.1)	29 (44.6)	16 (53.3)	45 (47.4)
Tail	10 (19.2)	9 (21.4)	19 (20.2)	10 (15.4)	2 (6.7)	12 (12.6)
Tumor biopsy site, n (%)						
Liver	40 (76.9)	23 (54.8)	63 (67.0)	39 (60.0)	18 (60.0)	57 (60.0)
Pancreas	6 (11.5)	9 (21.4)	15 (16.0)	10 (15.4)	7 (23.3)	17 (17.9)
Lung	1 (1.9)	2 (4.8)	3 (3.2)	2 (3.1)	1 (3.3)	3 (3.2)
Duodenum	1 (1.9)	1 (2.4)	2 (2.1)	2 (3.1)	0 (0.0)	2 (2.1)
Omentum	1 (1.9)	1 (2.4)	2 (2.1)	1 (1.5)	0 (0.0)	1 (1.1)
Lymph node	1 (1.9)	0 (0.0)	1 (1.1)	1 (1.5)	0 (0.0)	1 (1.1)
Abdomen	0 (0.0)	0 (0.0)	0 (0.0)	1 (1.5)	0 (0.0)	1 (1.1)
Abdominal cavity	0 (0.0)	0 (0.0)	0 (0.0)	1 (1.5)	0 (0.0)	1 (1.1)
Ampulla	0 (0.0)	0 (0.0)	0 (0.0)	1 (1.5)	0 (0.0)	1 (1.1)
Peritoneum	0 (0.0)	0 (0.0)	0 (0.0)	1 (1.5)	0 (0.0)	1 (1.1)
Papilla	0 (0.0)	0 (0.0)	0 (0.0)	0 (0.0)	1 (3.3)	1 (1.1)
History of diabetes, n (%)	18 (34.6)	18 (42.9)	36 (38.3)	19 (29.2)	13 (43.3)	32 (33.7)
C3M						
Mean	11.0	11.2	11.1	8.8	8.8	8.8
Range	15.7	28.5	28.5	13.0	9.5	13.2
Below LLOQ (< 1.9 ng/mL), No.	0	0	0	0	0	0
Above ULOQ (> 54.2 ng/mL), No.	0	0	0	0	0	0
PRO-C3						
Mean	32.2	35.7	33.7	27.8	24.1	26.7
Range	353.3	159.9	353.3	183.6	97.6	183.6
Below LLOQ (< 4 ng/mL), No.	0	0	0	0	0	0
Above ULOQ (> 107.6 ng/mL), No.	1	4	5	1	0	1
C3M/PRO-C3 ratio						
Mean	0.68	0.57	0.63	0.56	0.58	0.56
Range	1.73	2.29	2.29	2.13	1.74	2.13

Table shows demographics and clinical characteristics of patients for whom baseline plasma samples were available

Where range is provided as a single number, it represents the difference between the lowest and highest value

LLOQ: lower limit of quantification; ULOQ: upper limit of quantification

Biomarkers C3M, PRO-C3, and PRO-C6 were detectable in all samples (analyte above the lower limit of quantification), whereas C8-C and VCANM were detectable in only 33% and 37% of samples, respectively (Additional file 1: Fig. S2). Univariate Cox regression analyses using a median cut-off revealed no predictive value for the five individual ECM neo-peptide biomarkers in terms of PAG-specific PFS benefit in Stage 1 of the study (Table 2). However, a significant difference in PFS was observed in patients with a high C3M/PRO-C3 ratio, such that patients with a high C3M/PRO-C3 ratio had decreased risk of disease progression when treated with PAG compared with AG (HR 0.40; 95% CI 0.17–0.92; *P* = 0.031). In contrast, patients with a low C3M/PRO-C3 ratio had no significant difference in PFS between treatment arms (HR 1.09; 95% CI 0.55–2.18; *P* = 0.807).

**Table 2:** Effect of treatment for PAG compared with AG on PFS for the ECM biomarkers using the median cut-off point in stage 1 of HALO 109-202

Biomarker subgroup	HR	95% CI	*P*-value
C3M low	0.74	0.47–1.16	0.192
C3M high	0.70	0.33–1.45	0.332
C8-C^a^ low	0.72	0.38–1.38	0.323
C8-C^a^ high	0.56	0.21–1.45	0.231
PRO-C3 low	0.53	0.23–1.20	0.130
PRO-C3 high	0.88	0.43–1.80	0.735
PRO-C6 low	0.47	0.21–1.08	0.077
PRO-C6 high	1.07	0.53–2.17	0.853
VCANM^b^ low	0.63	0.32–1.23	0.176
VCANM^b^ high	0.73	0.30–1.74	0.725
C3M/PRO-C3 ratio low	1.09	0.55–2.18	0.807
C3M/PRO-C3 ratio high	0.40	0.17–0.92	0.031*

^a^Detectable in 33% of samples

^b^Detectable in 37% of samples

A univariate Cox regression analysis was used to calculate the predictive associations between biomarker levels and PFS. *A significant PFS benefit for PAG compared with AG was observed in the C3M/PRO-C3 ratio high subgroup (HR 0.40; 95% CI 0.17–0.92; *P* = .031). All other associations were non-significant

### The C3M/PRO-C3 ratio was predictive of PFS benefits for PAG compared with AG in stage 1

**Fig. 2:**
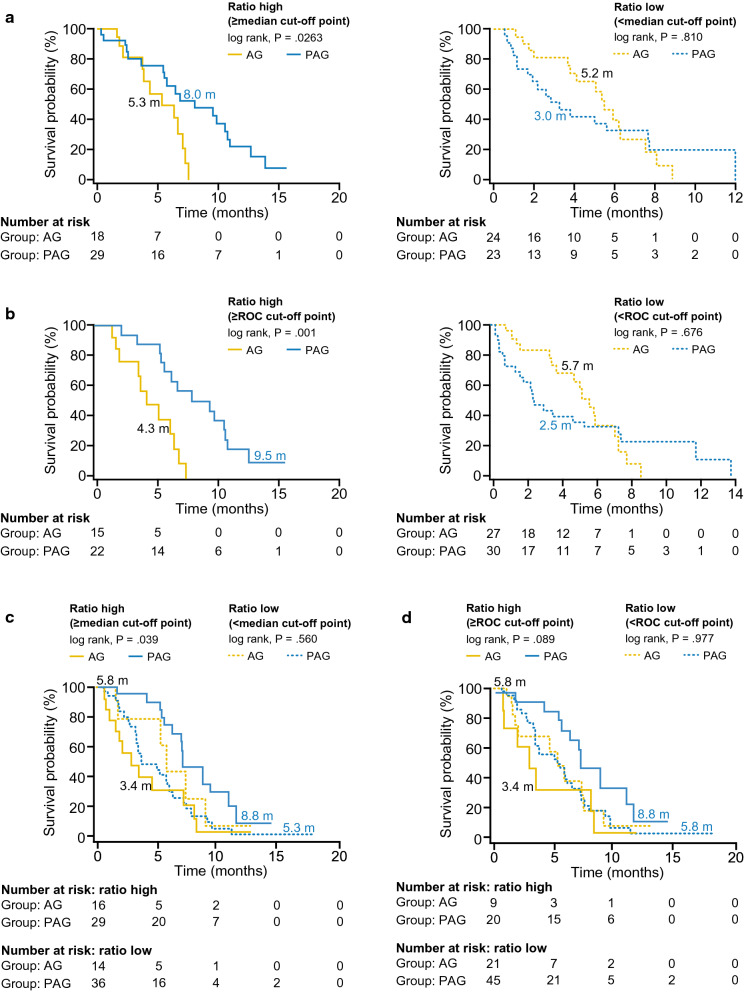
High C3M/PRO-C3 ratio was predictive of treatment specific PFS in Stage 1 (discovery) and Stage 2 (validation) of HALO 109-202. In Stage 1 (discovery), C3M/PRO-C3 ratio subgroups were defined by **a** median cut-off and **b** ROC cut-off. In Stage 2 (validation), C3M/PRO-C3 ratio subgroups were defined by **c** median cut-off derived from Stage 1 and **d** ROC cut-off derived from Stage 1

**Fig. 3:**
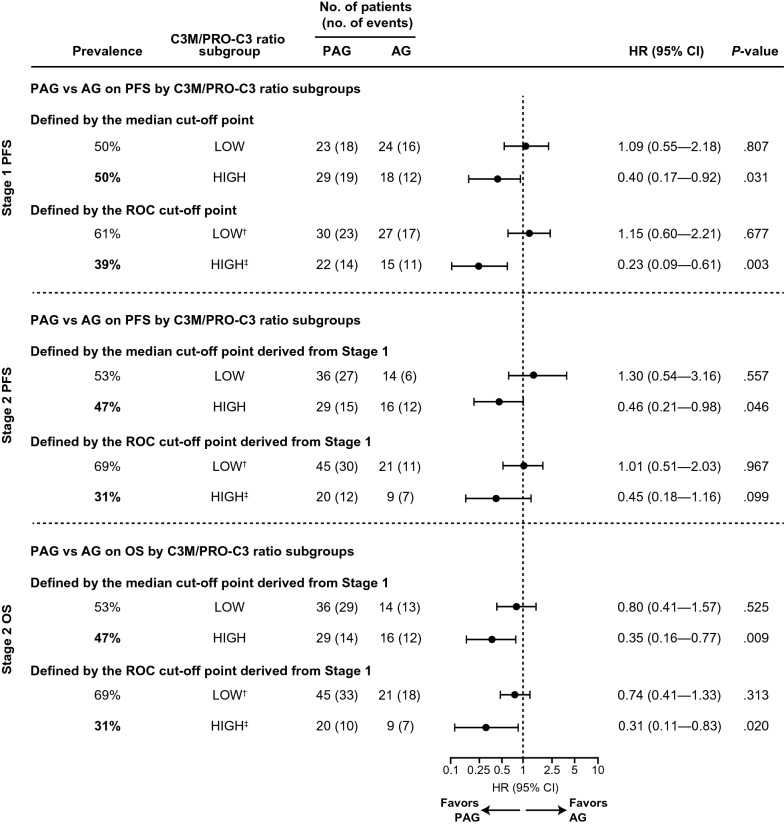
Forest plots of the effects of PAG versus AG in terms of PFS and OS by C3M/PRO-C3 ratio subgroups in Stage 1 and Stage 2 of HALO 109-202. In Stage 1, C3M/PRO-C3 ratio subgroups for PFS analysis were defined by median cut-off and ROC cut-off. In Stage 2, C3M/PRO-C3 ratio subgroups for PFS and OS analyses were defined by median cut-off derived from Stage 1 and ROC cut-off derived from Stage 1. Both median and ROC cut-offs for C3M/PRO-C3 ratio predicted a statistically significant benefit (P < .05) in PFS (Stage 1 and Stage 2) and OS (Stage 2) for PAG vs AG. ^†^ROC cut-off point from Stage 1 for the low biomarker subgroup was < 0.702. ^‡^ROC cut-off point from Stage 1 for the high biomarker subgroup was ≥ 0.702

Based on initial results, median and ROC cut-offs for the C3M/PRO-C3 ratio were further evaluated. A high C3M/PRO-C3 ratio predicted a significant PFS benefit for PAG compared with AG in Stage 1 using both median and ROC cut-offs. Using the median and ROC cut-offs, 50% and 39% of patients, respectively, were estimated to have a high C3M/PRO-C3 ratio. In patients with a high C3M/PRO-C3 ratio using the median cut-off, median PFS was 8.0 months with PAG treatment compared with 5.3 months with AG treatment (log-rank *P* = 0.0263; HR 0.40; 95% CI 0.17–0.92; *P* = 0.031; Figs. 2a, 3 and Table 2). A similar benefit was seen when high C3M/PRO-C3 ratio was defined using the ROC cut-off; patients treated with PAG had a median PFS of 9.5 months versus 4.3 months with AG (log-rank *P* = 0.001; HR 0.23; 95% CI 0.09–0.61; *P* = 0.003; Figs. 2b and 3). In patients with low C3M/PRO-C3 ratio, PFS was not significantly different between treatment arms using either cut-off (Figs. 2a and b, 3).

### C3M/PRO-C3 ratio was predictive of PFS and OS for PAG compared with AG in stage 2

Stage 2 plasma samples were used to validate ability of C3M/PRO-C3 ratio to predict response to PAG. Similar to Stage 1, 47% and 31% of patients in Stage 2 were estimated to have high C3M/PRO–C3 ratio using the Stage 1 median and ROC cut-offs, respectively.

The C3M/PRO-C3 ratio also predicted PFS benefit in Stage 2. In patients with high C3M/PRO-C3 ratio, PFS was longer with PAG than with AG (8.8 months versus 3.4 months) using the median cut-off (log-rank *P* = 0.039; HR 0.46; 95% CI  0.21–0.98; *P* = 0.046; Figs. 2c and 3). A trend towards significance for PFS improvement was observed with PAG versus AG using the ROC cut-off: 8.8 months versus 3.4 months (log-rank *P* = 0.089; HR 0.45; 95% CI 0.18–1.16; *P* = 0.099; Figs. 2d and 3d). As in Stage 1, PFS in patient with low C3M/PRO-C3 ratio was not significantly different for PAG compared with AG with either cut-off (Figs. 2a and b, 3).

**Fig. 4:**
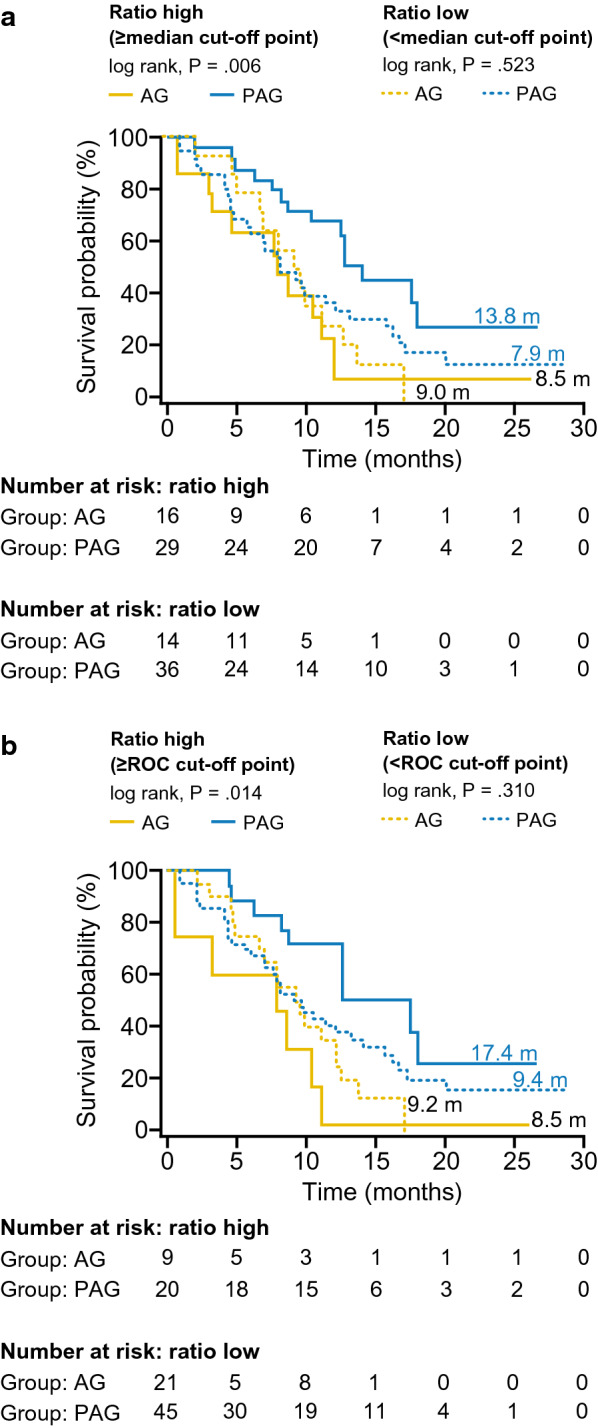
High C3M/PRO-C3 ratio was predictive of treatment-specific OS in Stage 2 of HALO 109-202 using Stage 1 cut-offs. C3M/PRO-C3 ratio subgroups defined by **a** median cut-off derived from Stage 1 and **b** ROC cut-off derived from Stage 1

High C3M/PRO-C3 ratio also predicted PAG treatment-specific OS benefit in Stage 2 using the cut-offs derived from Stage 1 (Stage 1 was not used to evaluate associations with OS as a relatively high proportion of these patients discontinued pegvorhyaluronidase alfa treatment at the time of the clinical hold [35]). With the median cut-off, OS was 13.8 months with PAG versus 8.5 months with AG (log-rank *P* = 0.006; HR 0.35; 95% CI 0.16–0.77; *P* = 0.009; Figs. 3 and 4a). With the ROC cut-off, OS was 17.4 months with PAG versus 8.5 months with AG (log-rank *P* = 0.014; HR 0.31; 95% CI 0.11–0.83; *P* = 0.020; Figs. 3 and 4b). There was no significant difference in OS in patients with low C3M/PRO-C3 ratio treated with PAG compared with AG using either median or ROC cut-offs (Figs. 3 and 4a and b). Thus, the C3M/PRO-C3 ratio cut-offs derived in Stage 1 were able to predict significant benefits in PFS and OS in PAG-treated patients with high C3M/PRO-C3 ratio for patients in Stage 2 of the study.

### Impact of C3M/PRO-C3 ratio on ORR

In patients with a high C3M/PRO-C3 ratio, the ORR was higher with PAG-compared with AG-treated patients in both Stage 1 (median cut-off, 55% versus 28%; ROC cut-off, 64% versus 20%) and Stage 2 (median cut-off, 52% versus 38%; ROC cut-off, 55% versus 33%; Additional file 1: Fig. S3).

### C3M/PRO-C3 ratio was predictive of PFS in patients with HA-high tumors and HA-low tumors

**Fig. 5:**
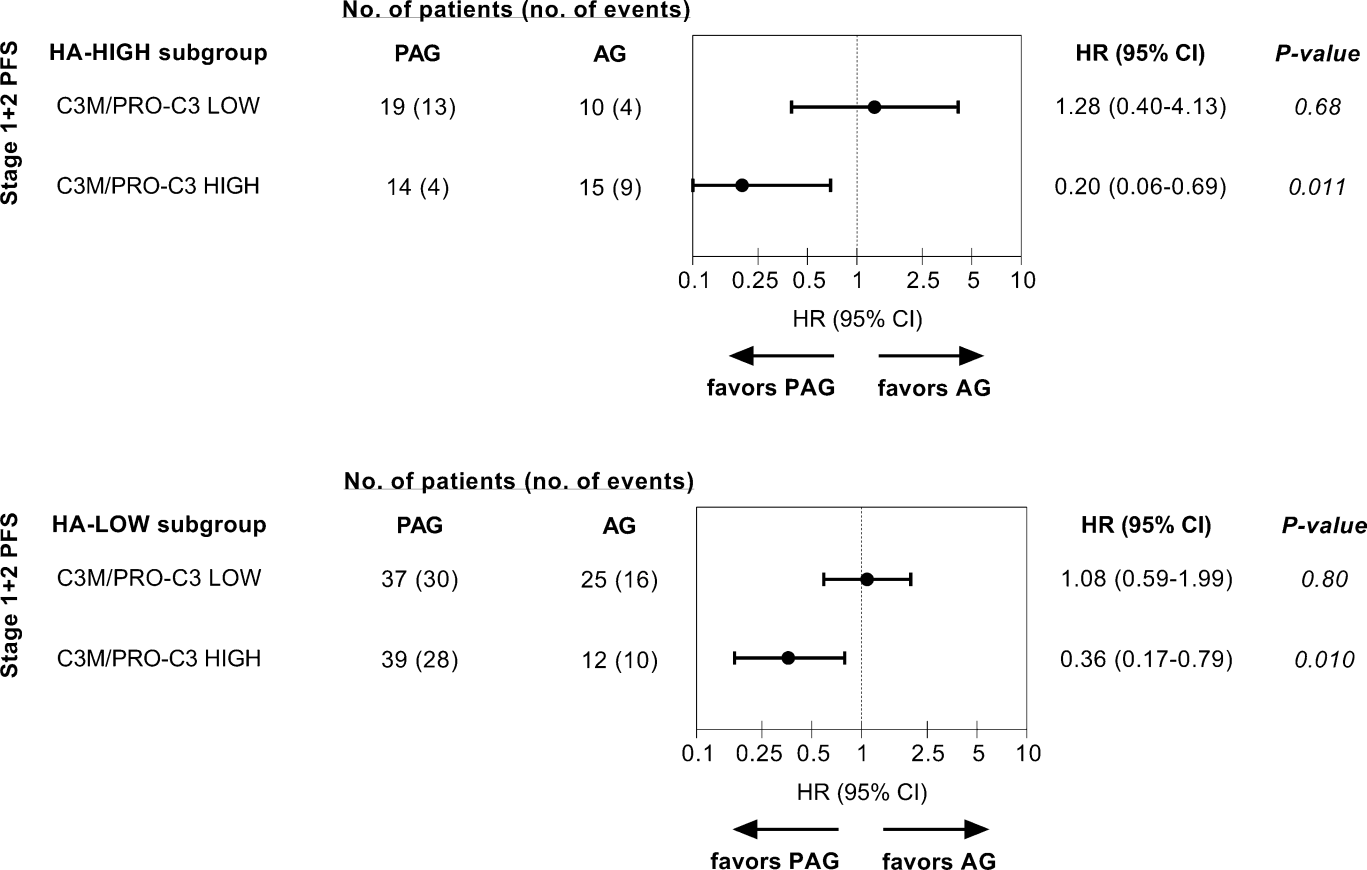
Forest plots of the effects of PAG versus AG in terms of PFS by C3M/PRO-C3 ratio in ‘HA-high’ and ‘HA-low’ subgroups in Stage 1 + 2 of HALO 109-202. C3M/PRO-C3 ratio subgroups were defined by the median cut-off from stage 1 in patients presenting with either HA-high (top) or HA-low (bottom) tumor biopsies measured with an affinity histochemistry assay previously described [35]

As mentioned, PFS improvement was previously shown to be more pronounced in HA-high tumors identified by an affinity histochemistry assay compared to HA-low tumors. Therefore, any potential overlap/add-on-effect for predicting response to PAG by combining HA tumor biopsy measures with the C3M/PRO-C3 plasma assay was evaluated. Interestingly, high C3M/PRO-C3 ratio (median cut-off) predicted for response to PAG in both HA-low and HA-high tumors, with the lowest HR for PAG vs AG being obtained in the subgroup of patients that were both HA-high and C3M/PRO-C3 high (HR 0.20; 95% CI 0.06–0.69; *P* = 0.011; Fig. 5).

## Discussion

In this retrospective analysis, circulating plasma biomarkers of ECM remodeling were investigated for their ability to predict survival outcomes of patients with mPDA treated with PAG versus AG. Based on univariate Cox regression analyses, pre-treatment levels of C3M, C8-C, PRO-C3, PRO-C6, and VCANM had no predictive value; however, high C3M/PRO-C3 ratio demonstrated utility as a biomarker to identify patients benefiting from PAG treatment.

The present study addresses a new, and non-invasive, biomarker approach/strategy to identify patients responding to PAG and is in contrast to the original report applying a tissue-based affinity histochemistry assay to identify patients with HA-high tumors [35]. Improvement in PFS for HA-high tumors (stage 1 + 2) treated with PAG vs AG was borderline significant with an HR 0.51; 95% CI 0.26–1.00; *P* = .048, and improvement in PFS for C3M/PRO-C3 ratio high tumors treated with PAG vs AG provided a HR 0.40; 95% CI 0.17–0.92; *P* = .031 (stage 1) and a HR 0.46; 95% CI 0.21–0.98; *P* = .026 (stage 2).

Our findings support previous data demonstrating the predictive/prognostic value of the C3M/PRO-C3 ratio in metastatic melanoma and pancreas cancer. In patients with metastatic melanoma, high PRO-C3 was predictive of poor OS with ipilimumab, whereas a high C3M/PRO-C3 ratio predicted increased OS [23]. The same observation occurred in advanced pancreas cancer patients treated with 5FU based therapy [30]. The mechanism underlying the predictive value of C3M and PRO-C3 remains unclear but is likely related to the excessive deposition of collagen found in the TME of mPDA [9]. Recent non–clinical data demonstrated a link between increasing HA content and elevated collagen and αSMA levels [15]. Thus, tumors with extremely high collagen deposition (and presumably increased HA content) may have reduced response to, or be less able to readily reverse, the HA degradation and ECM remodeling by pegvorhyaluronidase alfa. Unpublished data from our research group have shown that PRO-C3 is produced by pancreas cancer associated fibroblast, supporting these mechanisms discussed. It would therefore follow that tumors with a high C3M/PRO-C3 ratio, presumably with decreased collagen deposition (and reduced HA content), would be more likely to respond to, or have less capacity to reverse pegvorhyaluronidase alfa effects, resulting in a greater response. Regardless, the observation that a high C3M/PRO-C3 ratio predicts positive treatment outcomes underscores the importance of the balance between ECM degradation and formation and may have clinical relevance for guiding future biomarker development and evaluation of stromal modifiers in mPDA management.

Given the limited effective treatment options available for mPDA, predictive biomarkers represent a valuable tool for identifying potential patient subgroups most likely to respond to treatment. Moreover, patient subgroups deemed unlikely to benefit from a therapy based on presence or absence of a certain biomarker will be spared unnecessary treatment [44]. Predictive biomarkers can, therefore, enable clinicians to individually tailor therapeutic strategies to optimize outcomes. Several potential biomarkers have been identified that may predict response to different therapeutics in patients with pancreatic cancer. These include carboxylesterase 2 expression (neoadjuvant FOLFIRINOX), Hu-antigen R, microRNAs miR-142-5p and miR-204, human equilibrative nucleoside transporters and human concentrative nucleoside transporter-3 (gemcitabine), *BRCA* gene mutations (platinum-based chemotherapy and poly [Adenosine diphosphate ribose] polymerase inhibitors), and high microsatellite instability or mismatch repair deficiency (pembrolizumab) [44–52]. However, there remains a large gap between initial discovery and clinical translation for most of these potential biomarkers.

Translating promising research on biomarkers into clinically useful assays is a complex process. Current recommendations suggest that potential candidates undergo a multistep process involving discovery, verification in 100 or more patients, and validation in prospective trials [53]. The separate stages of HALO109-202 allowed us to use Stage 1 (n = 94) as the discovery/verification cohort, and Stage 2 (n = 95) as a validation cohort. With the halt of further development of pegvorhyaluronidase alfa, studies in larger cohorts with other stromal modifiers are required to expand on our findings. In-clinic assays based on minimally or non-invasive biomarkers are of particular value as they may encourage clinician use and patient compliance [38]. This approach may be appropriate when tissue biopsies are not possible or optimal. The C3M/PRO-C3 biomarker we identified is based on circulating plasma markers, providing an opportunity for a non-invasive liquid biopsy to evaluate patients who are more likely to respond to treatment. Furthermore, as high C3M/PRO-C3 ratio predicted for a PFS benefit to PAG vs. AG both in patients with HA-low tumors and HA-high tumors indicate that ECM/collagen turnover measured in plasma may provide additional value to a biopsy when applied for patient selection. Hence, the advantage of the liquid biopsy approach is that it can predict therapeutic benefits independently from a tissue-based biopsy and biomarker.

There remain some limitations to this analysis, including open-label nature of the study and the relatively small patient numbers in both cohorts, exacerbated by discontinuation rates during the temporary clinical hold. Moreover, the exploratory nature of this analysis may be prone to confounders, such as sex, age, stage, primary tumor site, and history of diabetes, potentially limiting interpretation of the results. Finally, C8-C and VCANM were detectable in only 33% and 37% of patients, respectively, perhaps limiting their analysis as biomarkers.

## Conclusion

We demonstrated and validated the potential of the C3M/PRO-C3 ratio as circulating plasma biomarkers, to identify patients with mPDA potentially benefiting from pegvorhyaluronidase alfa treatment when given in combination with chemotherapy (PAG). These findings warrant further validation of the C3M/PRO-C3 ratio as a predictive biomarker in large-scale mPDA studies. Additional research is needed to further elucidate the biological mechanism responsible for the observed predictive value of the C3M/PRO-C3 ratio and its applicability for other stromal modifiers.

## Supplementary information

**Additional file 1.** Supplementary Material

## Data Availability

Additional information about the studies and/or datasets can be obtained by contacting Halozyme Therapeutics, Inc. (11388 Sorrento Valley Road, San Diego, CA 92121, USA; Phone: +1.858.794.8889; Email: publications@halozyme.com) or Nordic Bioscience A/S (Herlev Hovedgade 205-207, 2730 Herlev, Denmark; Phone +4544525252; Email: mail@nordicbio.com)

## Abbreviations

AG: Nab-paclitaxel/gemcitabine
C3M: Matrix metalloprotease-mediated degradation of type III collagen
C8-C: C-terminal of type VIII collagen
ECM: Extracellular matrix
EDTA: K3-ethylenediaminetetraacetic acid
ELISA: Enzyme-linked immunosorbent assay
HA: Hyaluronan
HR: Hazard ratio
MMP: Matrix metalloprotease
mPDA: Metastatic pancreatic ductal adenocarcinoma
ORR: Objective response rage
OS: Overall survival
PAG: Pegvorhyaluronidase alfa plus nab-paclitaxel/gemcitabine
PDA: Pancreatic ductal adenocarcinoma
PEGPH20/PVHA: Pegvorhyaluronidase alfa
PFS: Progression free survival
PRO-C3: Released N-terminal pro-peptide of type III collagen
PRO-C6: C-terminal of released C5 domain of type VI collagen α3 chain
RNA: Ribonucleic acid
ROC: Receiver operating characteristics
TE: Thromboembolic
TME: Tumor microenvironment
VCANM: Matrix metalloprotease-mediated degradation of versican
αSMA: Alpha-smooth muscle actin
