# Supplementary material for: Blood-based extracellular matrix biomarkers as predictors of survival in patients with metastatic pancreatic ductal adenocarcinoma receiving pegvorhyaluronidase alfa
Source: J Transl Med. 2021 Jan 21;19:39. doi: 10.1186/s12967-021-02701-z (PMC7819178; doi:10.1186/s12967-021-02701-z)
Supplement: Supplementary file 1 — Additional file 1. Supplementary Material [file 12967_2021_2701_MOESM1_ESM.docx]

# Additional file

Table S1. Biomarker targets investigated

| **Biomarker** | **Description** |
| --- | --- |
| C3M | MMP-mediated degradation of type III collagen |
| C8-C | C-terminal of type VIII collagen |
| PRO-C3 | Released N-terminal pro-peptide of type III collagen |
| PRO-C6 | C-terminal of released C5 domain of type VI collagen α3 chain (endotrophin) |
| VCANM | MMP-mediated degradation of versican |

MMP, matrix metalloproteinase; VCANM, versican

Fig S1. Biology of the extracellular matrix.
PRO-C3 and PRO-C6 are involved in extracellular remodeling and collagen formation. C3M degrades collagen, and VCANM and C8-C are involved in repair.


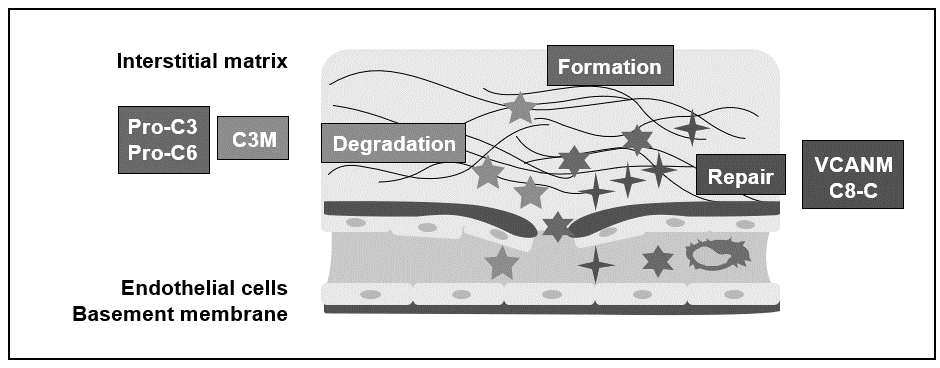


VACNM, versican

Fig S2. Biomarker scatter plots for biomarkers in the HALO 109-202 study.
Scatter plot analysis confirmed that biomarkers C3M, PRO-C3, and PRO-C6 were detectable in all samples from Stages 1 and 2 of the HALO 109-202 study. For VCANM, 63% of samples were below the LLOQ of 1.2 ng/mL and for C8-C, 67% of samples were below LLOQ of 1.4 ng/mL.


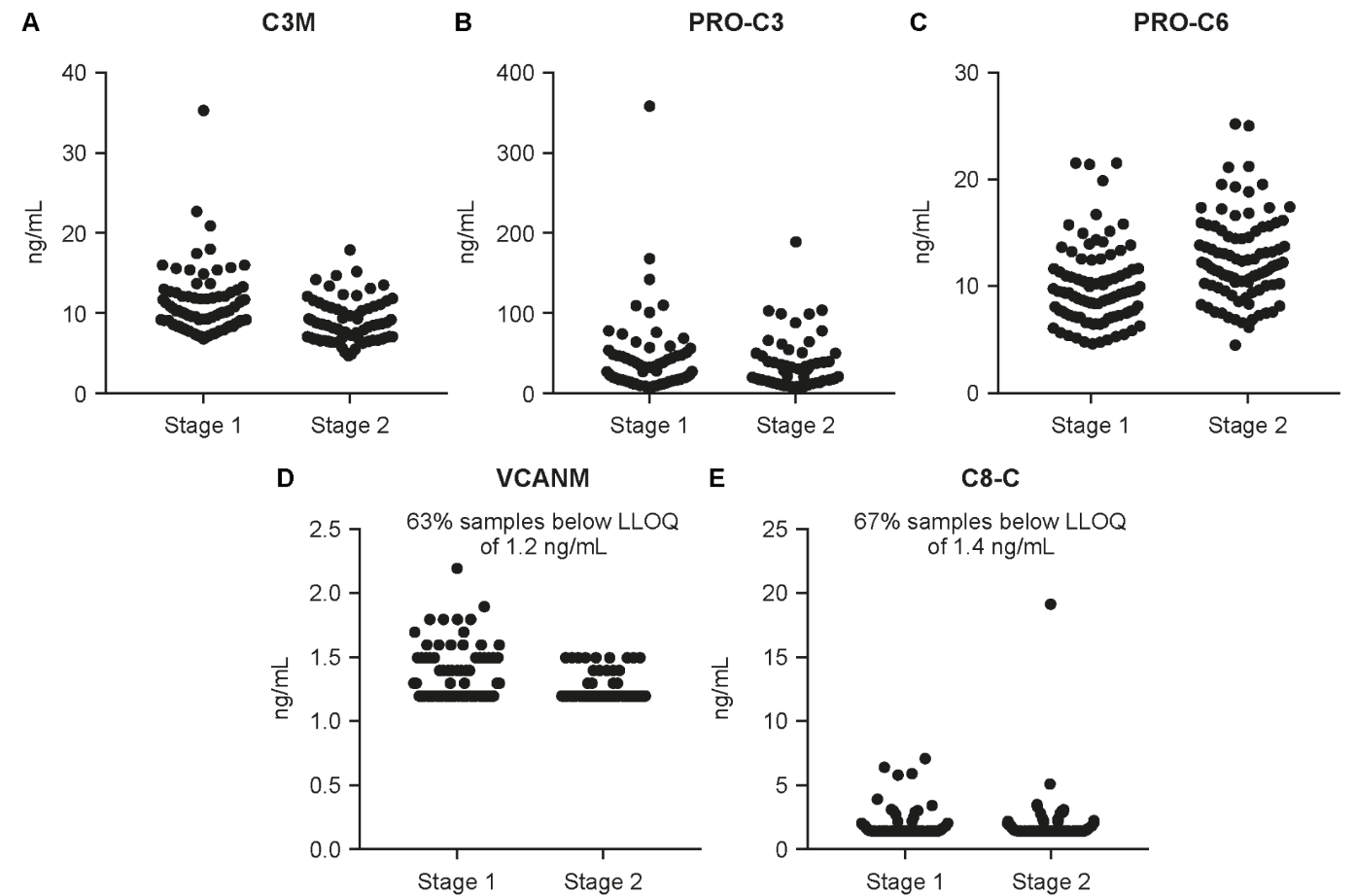


LLOQ, lower limit of quantitation; VACNM, versican

Fig S3. ORR of patients with high C3M/PRO-C3 ratio.
ORR of patients with high In Stage 1 of HALO 109-202, C3M/PRO-C3 ratio was defined by (A) median and (B) ROC cut-offs. In Stage 2 of HALO 109-202, C3M/PRO-C3 ratio was defined by (C) median and (D) ROC cut-offs derived from Stage 1 of HALO 109-202. The response data and ORR were based on unconfirmed responses and were analyzed descriptively.


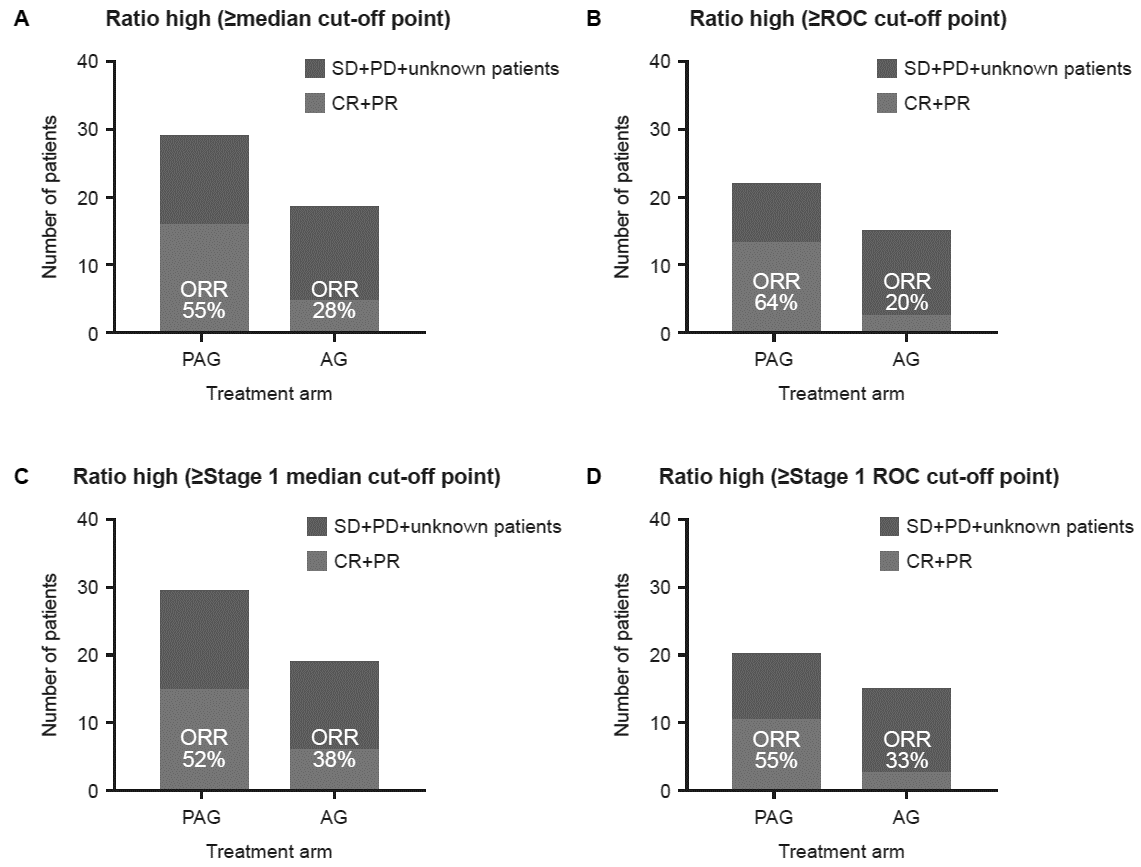
 AG, nab-paclitaxel + gemcitabine; CR, complete response; PAG, pegvorhyaluronidase alfa + nab-paclitaxel + gemcitabine; ORR, objective response rate; PD, progressive disease; PR, partial response; ROC, receiver-operating characteristic; SD, stable disease.
